# Diving Into the Rarity: A Case Report of Nonsyndromic Bilateral Branchial Cleft Fistula

**DOI:** 10.1002/ccr3.71403

**Published:** 2025-11-20

**Authors:** Rasmita Poudel, Anish Luitel, Shiv Bhushan Pandit, Bipin Koirala, Rahul Kumar Mahato, Aahana Pokharel, Ashish Raj Bista, Surya Bahadur Parajuli

**Affiliations:** ^1^ Birat Medical College Teaching Hospital Morang Nepal; ^2^ Department of Otorhinolaryngology Birat Medical College Teaching Hospital Morang Nepal; ^3^ Department of Community Medicine Birat Medical College Teaching Hospital Morang Nepal

**Keywords:** branchial, case report, fistula, nonsyndromic, pharyngeal arch

## Abstract

Bilateral branchial cleft fistulas are extremely rare congenital anomalies that may be part of a larger syndromic association. Prompt recognition and treatment are crucial in syndromic patients to halt disease progression, improve outcomes, and reduce the risk of long‐term complications. Complete surgical excision is the standard treatment for fistulas.

## 1 Introduction

Branchial cleft anomalies are the second most common congenital head and neck anomalies in children. They result from the incomplete obliteration of the branchial cleft during embryogenesis and typically present as cysts, sinus tracts, or fistulae in the anterolateral aspect of the neck [1]. Anomalies arising from the second branchial arch account for approximately 95% of cases [2]. Most branchial cleft anomalies are unilateral, while bilateral presentations are rare, comprising only about 1% of cases [1]. In most instances, bilateral branchial cleft fistulae are associated with branchiootorenal (BOR) syndrome, making the identification of syndromic features critical for proper evaluation and management [3]. Here, we report a rare case of a female child with bilateral branchial cleft fistula without any syndromic association, who underwent complete surgical excision and remains well on follow‐up.

## 2 Case Report

### 2.1 History and Examination

A 3‐year‐old female child, accompanied by her mother, presented to the Otorhinolaryngology OPD of our hospital with a chief complaint of swelling on the right lower anterior part of the neck. The swelling was painless and associated with spontaneous discharge, which was scanty, purulent, non‐foul‐smelling, and non‐blood‐stained. Her mother gives a history of similar episodes of swelling and discharge either from the right or left anterolateral lower third of the neck since 4 months of life. Swelling and discharge are aggravated during episodes of upper respiratory tract infection. However, there is no history of passage of food and water during swallowing. There is no history of fever, nausea, vomiting, regurgitation of food, or painful jaw movement. Similarly, there are no complaints of hearing difficulty, ear discharge, nasal discharge, painful swallowing, cough, failure to thrive, or weight loss. There is no history of tuberculosis. There is no history of hearing abnormalities or renal anomalies among family members. Her perinatal and developmental history is unremarkable.

**FIGURE 1:**
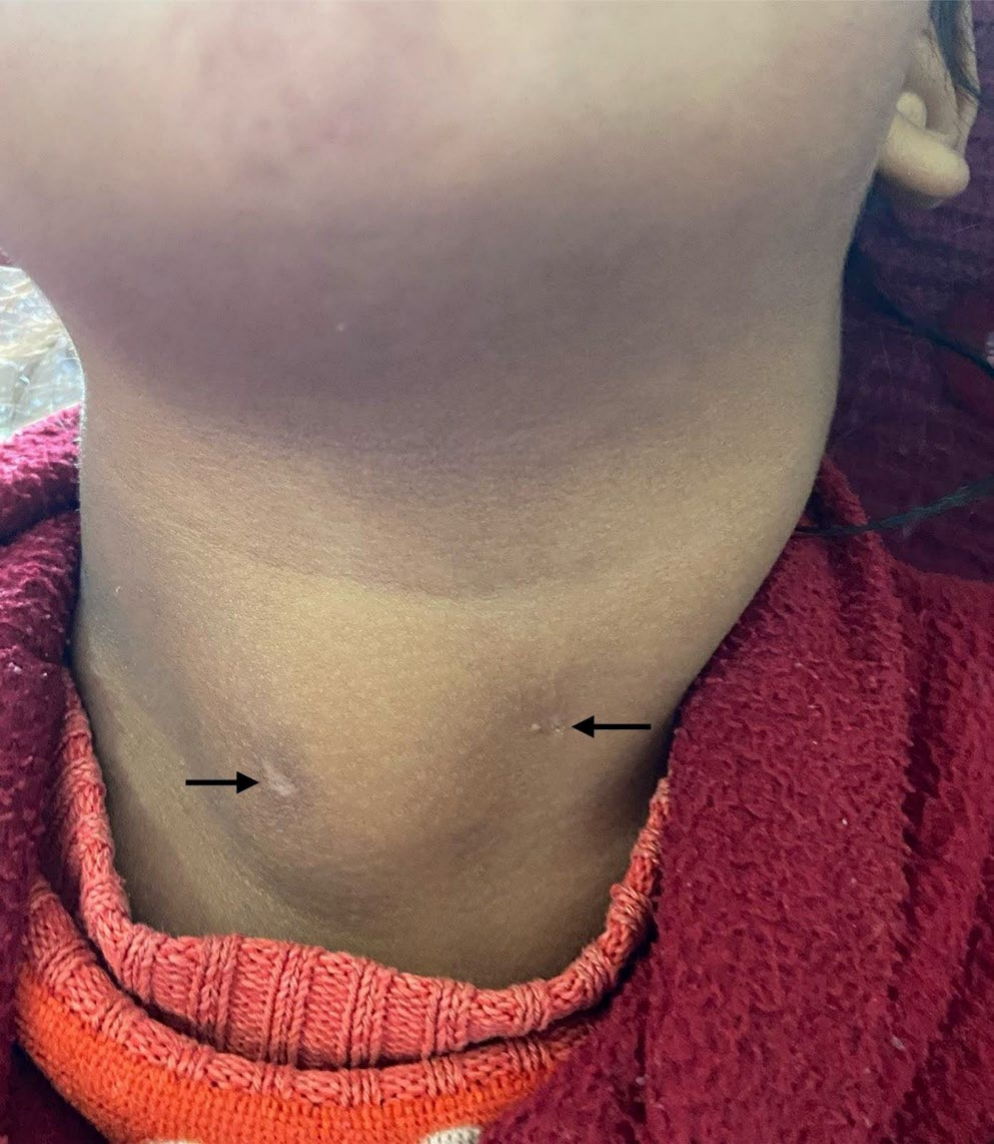
Preoperative photograph of the child showing bilateral pinpoint fistula openings (back arrows). Notice the swelling from the underlying cyst on the right side.

On clinical examination, the child appeared alert, active, and playful. Vital signs were within normal limits, and systemic examination did not reveal any abnormalities. Local examination of the neck demonstrated bilateral pinpoint cutaneous openings located at the junction of the upper two‐thirds and lower one‐third of the anterior border of the sternocleidomastoid muscle (Figure 1). A 3 × 3 cm non‐tender, smooth, fluctuant swelling with purulent discharge was noted on the right anterolateral aspect of the neck. The overlying skin was intact with no erythema or other dermatologic changes. There were no associated cutaneous lesions along the suspected fistulous tract. No preauricular or postauricular pits or sinuses were observed. Examination of the oral cavity was normal.

### 2.2 Investigation and Treatment

She had undergone non‐contrast computed tomography (NCCT) followed by contrast‐enhanced computed tomography (CECT) of the neck in another center, which showed a well‐defined fluid attenuation lesion measuring 21 mm × 12 mm approximately in the right supraclavicular region, anterior to the sternocleidomastoid muscle(SCM). In addition, small tracts were seen to continue from the above‐mentioned lesions, extending to the posterior aspect of the right submandibular gland. Along with this, there was a non‐enhancing fibrous tract from the subcutaneous plane anterior to the left SCM extending to the posterior aspect of the left submandibular gland.

We performed an ultrasonography neck, which demonstrated a thick‐walled cystic lesion measuring about 2.4 cm x 0.9 cm with echogenic content at the right supraclavicular region, anterior to and along the SCM and right thyroid lobe extending to the posterior aspect of the right submandibular gland. A diagnosis of bilateral branchial cleft fistula was made, where the right branchial cleft fistula was associated with the right branchial cleft cyst.

**FIGURE 2:**
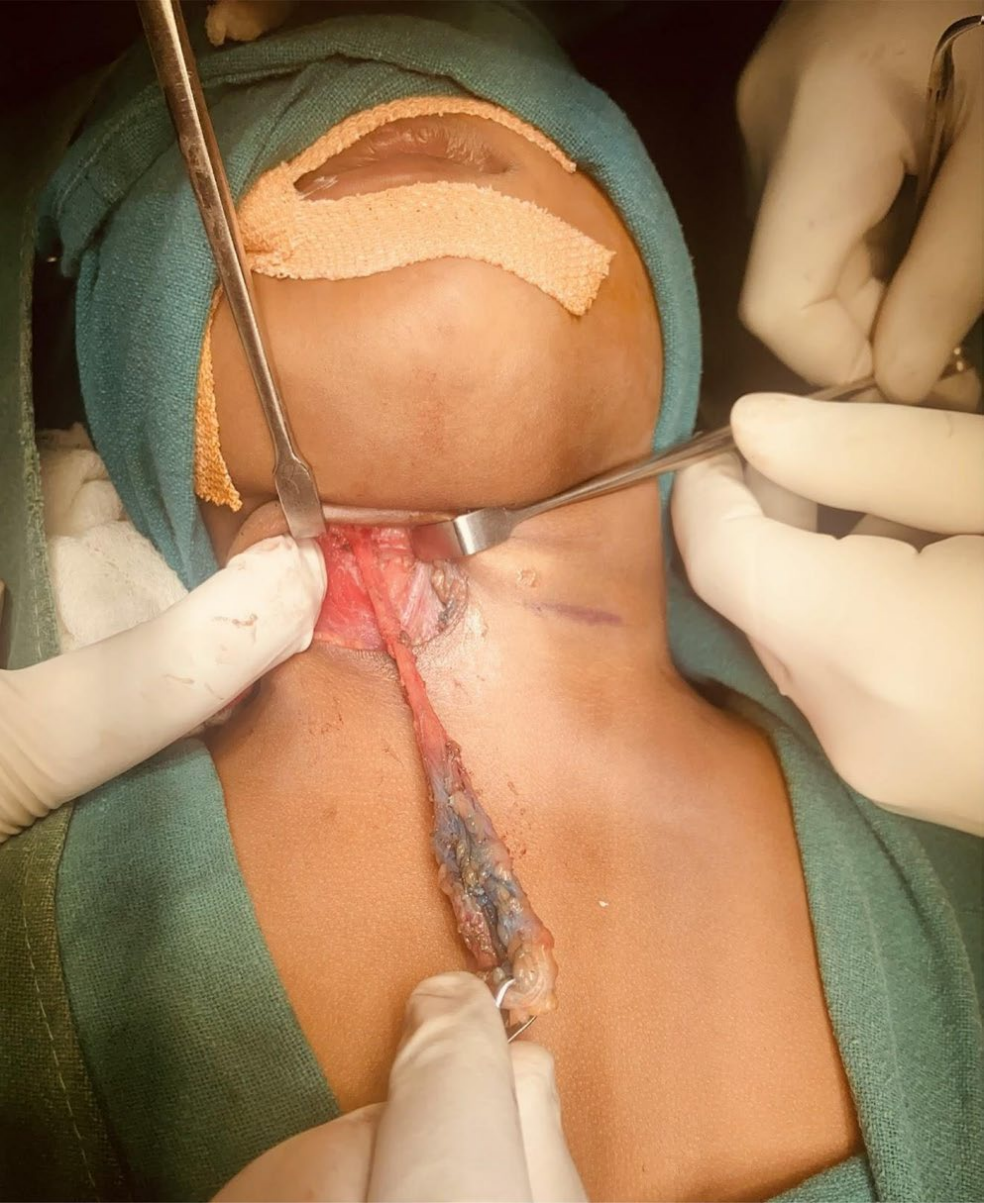
Intraoperative photograph showing excision of right branchial fistula tract using a single transverse incision. Blue tinge is of Methylene blue dye used to delineate the tract.

**FIGURE 3:**
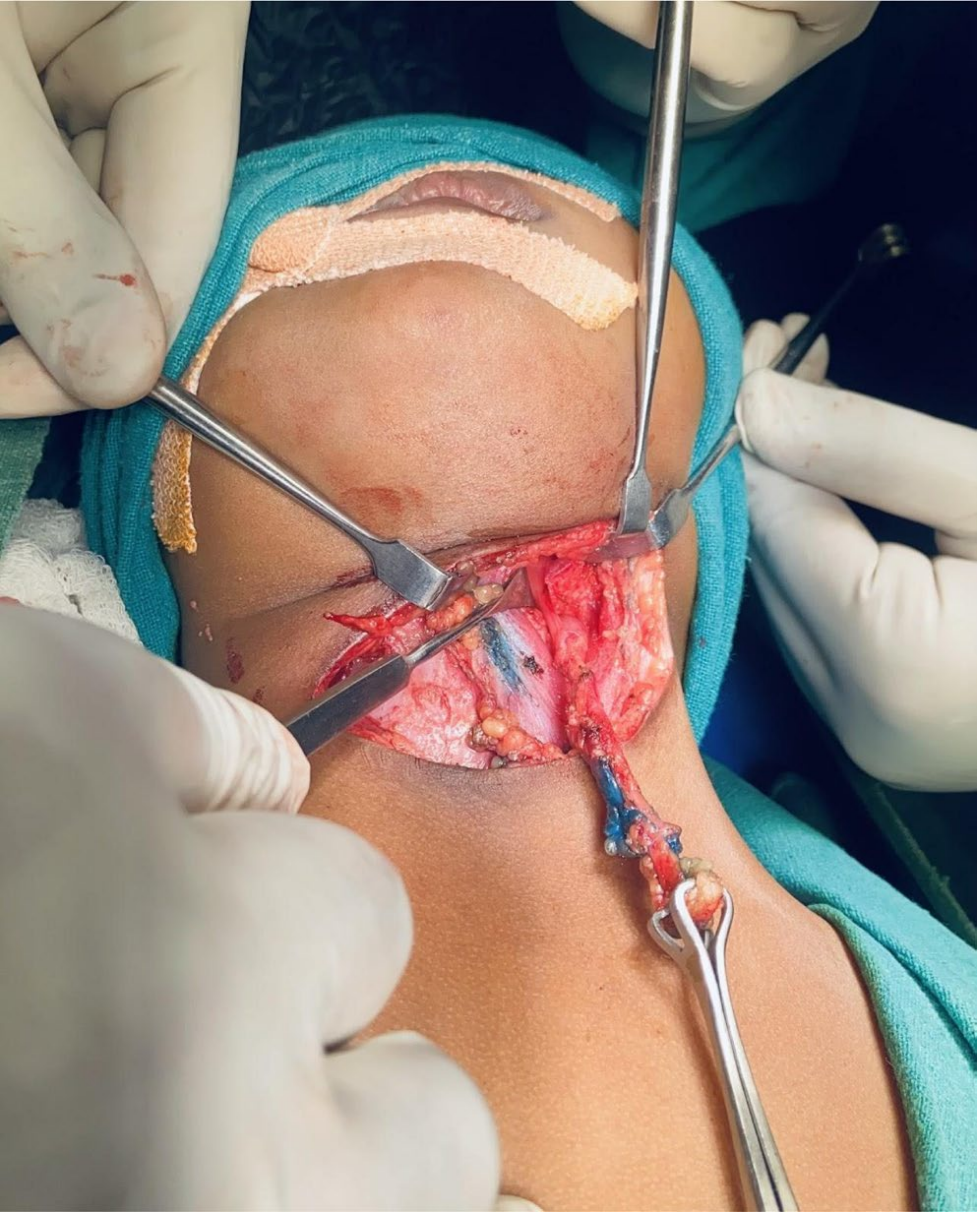
Intraoperative photograph showing excision of left branchial fistula tract using a single transverse incision. Blue tinge is of Methylene blue dye used to delineate the tract.

Following preoperative investigations and anesthesia fitness clearance, we planned excision of bilateral fistula tracts in a single surgical setting using a single transverse incision. This approach was selected due to the short length of the tracts and the surgeons being familiar with the technique. Under general anesthesia, with the patient in a supine position and the neck slightly extended, a single transverse cervical incision was made approximately 2 cm above the medial ends of the clavicles, extending from the anterior border of the right sternocleidomastoid muscle to the left. On the right side, the incision was deepened through the subcutaneous tissue and platysma. Upon identifying the surface plane of the right branchial cyst, it was carefully dissected from surrounding tissues. Then, its superior end was traced to excise the fistula. The tract was traced upward using methylene blue dye. Occasionally, we used a probe to guide the tract. It was found to pass between the bifurcation of the common carotid artery and ascend toward the tonsillar fossa. The tract was ligated just below its internal opening, which was left in situ (Figure 2). The left tract was dissected and excised using the same technique (Figure 3). Hemostasis was achieved, and the incision was closed in layers. Gross examination revealed the right tract measured 4.8 cm and the left 4.9 cm. Both specimens were sent for histopathological analysis.

## 3 Results (Outcome and Follow‐up)

**FIGURE 4:**
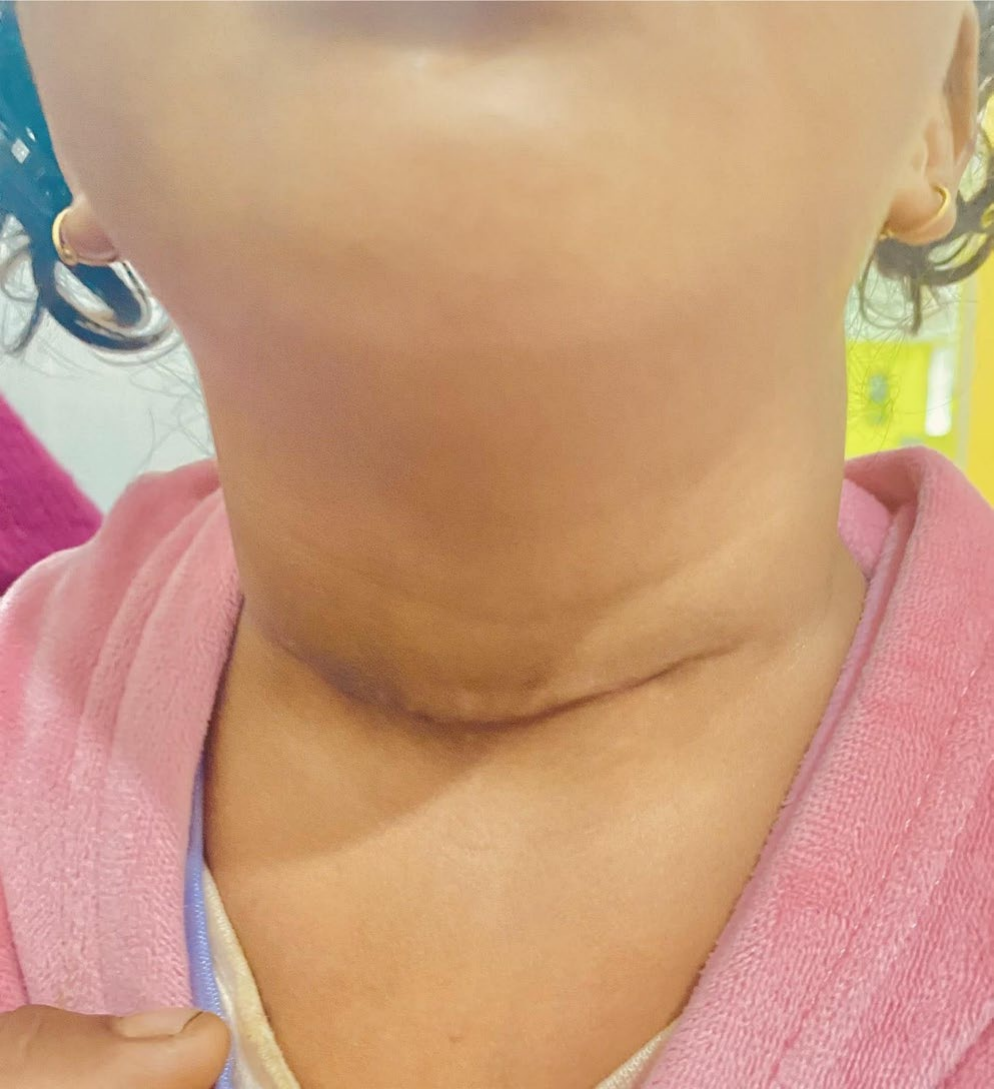
Postoperative photograph showing well‐healed transverse scar on neck.

The patient's immediate postoperative recovery was uneventful. No complications such as hematoma, infection, or nerve injury were observed. She was discharged in stable condition on the third postoperative day. Histopathological examination of both excised fistula tracts revealed respiratory‐type epithelium with few goblet cells. The underlying fibrotic wall demonstrated the presence of scattered polymorphonuclear leukocytes and well‐formed lymphoid follicles with prominent germinal centers. These histological features are consistent with a diagnosis of branchial cleft fistula. No evidence of atypical or malignant cells was seen. The patient was followed up regularly for 1 year postoperatively. During this period, there were no clinical signs of recurrence, and the surgical site healed well with satisfactory cosmetic and functional outcomes (Figure 4).

## 4 Discussion

Pharyngeal arch, previously known as branchial arch because of its resemblance to fish gills, is a bar of mesenchymal tissue that develops on either side of the embryonic head and neck [4]. Each arch has its own muscular component, supplied by its own artery and innervated by cranial nerves [2]. It is covered internally by endoderm while externally by surface ectoderm. Branchial arch abnormality results from incomplete obliteration of clefts and pouches.

Anomaly of the second branchial arch is the most common, followed by the first branchial arch anomaly. Anomalies of the third and fourth arches are rare. Branchial anomalies may occur as a part of other syndromes, particularly branchiootorenal syndrome. It is a rare autosomal dominant disorder caused by an EYA1 mutation [5]. As the name suggests, it is characterized by branchial cleft anomalies (sinus, cyst, fistula), otologic anomalies which may affect the external ear (e.g., preauricular pits, skin tags), middle ear or inner ear (e.g., hearing loss which can be conductive, sensorineural or mixed type; hypoplasia of semicircular canals) and renal malformations like agenesis, hypoplasia and dysplasia [3, 6]. In our case, there are no facial deformities, hearing loss, or renal impairment. Furthermore, there is no family history of hearing impairment or renal impairment, making a syndromic association unlikely.

Treatment of branchial cleft sinus and fistula is important for many reasons. First and foremost is its likelihood of getting infected repeatedly, leading to pus discharge, abscess formation, and pain. Local complications from the spread of infection to adjacent structures can cause dysphagia, dysphonia, stridor, dyspnea, and sometimes it may track circumferentially to cause a fatal retropharyngeal abscess [7]. However, malignant transformation of the cyst is rare [8, 9]. Our case was uncomplicated, presenting only with swelling and discharge.

The diagnosis of a branchial fistula is often straightforward based on clinical history and physical examination. However, additional imaging studies may be necessary to exclude associated anomalies, delineate the extent of the fistulous tract, and assist in surgical planning. Ultrasonography is a cost‐effective and widely accessible modality that can differentiate between solid and cystic components of the lesion and evaluate for concurrent renal anomalies. Advanced imaging techniques such as computed tomography (CT), magnetic resonance imaging (MRI), and fistulography provide detailed visualization of the fistulous tract, its course, and potential connections, thereby facilitating complete surgical excision and hence avoiding the rate of recurrence [10, 11].

Surgery is the definitive treatment of choice for a branchial fistula. It can be achieved through an open technique or endoscope‐assisted resection. Open resection of fistula is a traditional method of choice and still a widely performed procedure. With the evolution of minimally invasive surgery, endoscope‐assisted techniques are also gaining widespread popularity.

In the open technique for branchial fistula resection, two surgical approaches are in practice: the transcervical approach, which may involve either a single incision or a step‐ladder incision, and the combined transcervical and intraoral approach [10]. The excision of a fistula typically begins at the external opening, progressing in a cephalad direction (upward) until it reaches the internal opening in the tonsillar fossa, where the base of the fistula is excised. In cases where the fistula tract is longer or more complex, a second incision may be required higher up. This is known as a step‐ladder incision, a technique discovered by Bailey in 1993. This approach involves making two incisions in the neck to provide better exposure of the fistula tract [12]. Surgeons should keep in mind that the higher incisions need to be larger, as the fistula tract tends to be deeper in this region. Additionally, vital neurovascular structures are more closely located to the tract, so a larger incision ensures better visibility and safer dissection [13]. In the combined approach, the elliptical area of mucosa around the internal opening in the tonsillar fossa is excised [14]. There is a slightly higher risk of recurrence with the transcervical approach alone compared to the combined approach, probably because of incomplete resection of the tract in the former. No recurrence is found with the combined approach [10]. In cases with a short fistulous tract, as in our patient, complete surgical excision can be achieved through a single incision approach. Complete excision of the tract is essential to prevent recurrence [15]. Anatomically, the fistulous tract typically courses between the bifurcation of the carotid artery, ascending laterally to the glossopharyngeal and hypoglossal nerves, and terminating at the posterior tonsillar pillar. In our case, methylene blue dye was utilized to aid in delineating the tract. However, literature suggests that the use of methylene blue dye is not essential for the successful dissection of the branchial cleft fistula [11]. Additionally, tonsillectomy is generally not recommended.

In the newer endoscopic techniques, two primary approaches are commonly employed in practice: The anterior chest approach and the retroauricular approach [16]. These techniques conceal the surgical scar—either below the clavicle in the anterior chest approach or behind the ear in the retroauricular approach. They are designed to optimize cosmetic outcomes by avoiding visible scarring in the cervical region. During dissection around the fistulous tract, critical anatomical structures such as the carotid arteries and cranial nerves—including the vagus, hypoglossal, and glossopharyngeal nerves—are in close proximity, necessitating precise and meticulous dissection. Consequently, these procedures require a high level of surgical expertise and a thorough understanding of the endoscopic anatomy of the neck [17]. Moreover, intraoperative complications such as hemorrhage can be challenging to manage using endoscopic techniques alone, and there remains a potential need for conversion to an open surgical approach if necessary. Overall, it has been estimated that the recurrence rate of open surgery is about 3%–7% [18, 19]. In a study conducted by Xiao et al., where they operated on four patients using the anterior chest method, none of them had encountered any complications or recurrences, thus strengthening the evidence of safe excision using the endoscope‐assisted method [17].

Owing to the rarity of the case and the paucity of literature, we believe that further evidence on safety and recurrences following endoscopic techniques will come into the literature in the future.

## 5 Conclusion

Bilateral branchial cleft fistula is a rare congenital anomaly. It is important to evaluate possible syndromic associations, particularly Branchio–Oto–Renal (BOR) syndrome, as the management of associated anomalies is essential to prevent further complications. Diagnosis can typically be made based on a detailed history and physical examination. However, imaging studies such as a CT fistulogram are valuable for delineating the fistula tract and aiding in surgical planning. In our case, the fistula was nonsyndromic, and complete surgical excision of the tract followed by regular postoperative follow‐up resulted in an excellent outcome.

## Author Contributions

**Rasmita Poudel:** conceptualization, writing – original draft, writing – review and editing. **Anish Luitel:** conceptualization, writing – original draft, writing – review and editing. **Rahul Kumar Mahato:** supervision, writing – original draft, writing – review and editing. **Ashish Raj Bista:** writing – original draft, writing – review and editing. **Aahana Pokharel:** writing – original draft, writing – review and editing. **Surya Bahadur Parajuli:** supervision, writing – review and editing. **Bipin Koirala:** supervision, writing – review and editing. **Shiv Bhushan Pandit:** supervision, writing – review and editing.

## Ethics Statement

The authors have nothing to report.

## Consent

Informed, well‐written consent was obtained from the patient's parents for publication.

## Conflicts of Interest

The authors declare no conflicts of interest.

## Data Availability

Data generated/analyzed during this study is available from the corresponding author on reasonable request.

## References

[1] Y. Bajaj , S. Ifeacho , D. Tweedie , et al., “Branchial Anomalies in Children,” International Journal of Pediatric Otorhinolaryngology 75, no. 8 (2011): 1020–1023, https://pubmed.ncbi.nlm.nih.gov/21680029/. 21680029 10.1016/j.ijporl.2011.05.008

[2] A. Adams , K. Mankad , C. Offiah , and L. Childs , “Branchial Cleft Anomalies: A Pictorial Review of Embryological Development and Spectrum of Imaging Findings,” Insights Into Imaging 7, no. 1 (2016): 69, https://www.ncbi.nlm.nih.gov/pmc/articles/PMC4729717/. 26661849 10.1007/s13244-015-0454-5 PMC4729717

[3] C. P. Worden , K. C. Michaels , and W. P. Magdycz , “Nonsyndromic bilateral second branchial cleft fistulae: A case report,” Otolaryngology Case Reports 20 (2021): 100308, 10.1016/j.xocr.2021.100308.

[4] J. Casale and A. O. Giwa , Embryology, Branchial Arches (StatPearls Publishing, 2025), https://www.ncbi.nlm.nih.gov/books/NBK538487/. 30860722

[5] K. D. Klingbeil , C. M. Greenland , S. Arslan , et al., “Novel EYA1 Variants Causing Branchio‐oto‐Renal Syndrome,” International Journal of Pediatric Otorhinolaryngology 98 (2017): 59–63, 10.1016/j.ijporl.2017.04.037. 28583505 PMC5516569

[6] S. B. Nasir , S. J. Ladan , A. N. Bemu , and J. Jibrin , “Branchiootorenal Syndrome: A Case Report,” Nigerian Postgraduate Medical Journal 25, no. 1 (2018): 60–62, https://pubmed.ncbi.nlm.nih.gov/29676348/. 29676348 10.4103/npmj.npmj_203_17

[7] N. A. Salahuddin , S. S. Sanmugam , A. B. Az , et al., “Non‐Syndromic Bilateral Branchial Cyst: A Case Report,” Oman Medical Journal 38, no. 3 (2023): e515, https://pubmed.ncbi.nlm.nih.gov/37313249/. 37313249 10.5001/omj.2023.25 PMC10258544

[8] A. E. Sutton and J. Goldman , Branchial Cleft Cysts (StatPearls Publishing, 2025), https://www.ncbi.nlm.nih.gov/books/NBK482467/. 29494074

[9] M. Bellakhdhar , W. El Abed , S. Mestiri , S. Majdoub , W. Kermani , and M. Abdelkefi , “Malignant Degeneration of a Branchial Cyst: A Case Report,” Journal of Stomatology, Oral and Maxillofacial Surgery 119, no. 5 (2018): 440–443, 10.1016/j.jormas.2018.04.002. 29679737

[10] S. Sahu , A. Kumar , and T. S. Ramakrishnan , “Branchial Fistula: An Imaging Perspective,” Armed Forces Medical Journal India 67, no. 3 (2011): 262, https://www.ncbi.nlm.nih.gov/pmc/articles/PMC4920829/. 10.1016/S0377-1237(11)60056-7 PMC4920829 27365819

[11] K. N. Rattan , S. Rattan , D. Parihar , J. S. Gulia , and S. P. S. Yadav , “Second Branchial Cleft Fistula: Is Fistulogram Necessary for Complete Excision,” International Journal of Pediatric Otorhinolaryngology 70, no. 6 (2006): 1027–1030, 10.1016/j.ijporl.2005.10.014. 16343647

[12] A. D. Olusesi , “Combined Approach Branchial Sinusectomy: A New Technique for Excision of Second Branchial Cleft Sinus,” Journal of Laryngology and Otology 123, no. 10 (2009): 1166–1168, 10.1017/S0022215109990405. 19566978

[13] S. A. Patigaroo , W. u. Hamid , S. Ahmed , N. H. Dar , S. A. Showkat , and M. A. Latoo , “Complete Second Branchial Cleft Fistulas: A Clinicosurgical Experience,” Indian Journal of Otolaryngology and Head & Neck Surgery 75, no. 3 (2023): 1517, https://pmc.ncbi.nlm.nih.gov/articles/PMC10447783/. 37636759 10.1007/s12070-023-03565-z PMC10447783

[14] P. R. De and T. Mikhail , “A Combined Approach Excision of Branchial Fistula,” Journal of Laryngology and Otology 109, no. 10 (1995): 999–1000, https://pubmed.ncbi.nlm.nih.gov/7499958/. 7499958 10.1017/s002221510013186x

[15] L. C. Goh , R. S. Norain , Z. Shifa , and A. M. Manuel , “Bilateral Second Arch Branchial Fistula‐A Case Report,” Iranian Journal of Otorhinolaryngology 31, no. 107 (2019): 383, https://www.ncbi.nlm.nih.gov/pmc/articles/PMC6914326/. 31857983 10.22038/ijorl.2019.35910.2186 PMC6914326

[16] S. Dutta , B. Slater , M. Butler , and C. T. Albanese , ““Stealth Surgery”: Transaxillary Subcutaneous Endoscopic Excision of Benign Neck Lesions,” Journal of Pediatric Surgery 43, no. 11 (2008): 2070–2074, https://pubmed.ncbi.nlm.nih.gov/18970942/. 18970942 10.1016/j.jpedsurg.2008.03.031

[17] P. Han , J. Wang , F. Liang , P. Lin , R. Chen , and X. Huang , “Endoscope‐Assisted Resection of Second Branchial Cleft Fistula via the Anterior Chest Approach,” World Journal of Otorhinolaryngology ‐ Head and Neck Surgery 11, no. 3 (2024): 406, https://pmc.ncbi.nlm.nih.gov/articles/PMC12418354/. 40932913 10.1002/wjo2.227 PMC12418354

[18] A. C. Holt , D. H. Lofgren , and C. Shermetaro , Branchial Cleft Anomalies (StatPearls Publishing, 2025), https://www.ncbi.nlm.nih.gov/pubmed/29763089. 29763089

[19] D. H. Lee , T. M. Yoon , J. K. Lee , and S. C. Lim , “Clinical Study of Second Branchial Cleft Anomalies,” Journal of Craniofacial Surgery 29, no. 6 (2018): e557–e560, https://journals.lww.com/00001665‐201809000‐00087. 29608472 10.1097/SCS.0000000000004540

